# Temperature-Dependent Electrical Characteristics of Silicon Biristor

**DOI:** 10.3390/mi14122165

**Published:** 2023-11-28

**Authors:** Eunseong Kim, Doohyeok Lim

**Affiliations:** School of Electronic Engineering, Kyonggi University, Suwon 16227, Republic of Korea

**Keywords:** biristor, bistable resistor, temperature-dependent, positive feedback, negative feedback

## Abstract

In this study, we investigate the temperature-dependent electrical characteristics of bistable silicon resistors (biristors) at temperatures ranging from 275 to 400 K. The proposed biristor exhibits low latch voltages owing to the surface accumulation layer transistor concept. Moreover, the biristor was abruptly turned on and off by positive and negative feedback phenomena, respectively. As the temperature increased from 275 to 400 K, the latch-up voltage decreased from 2.131 to 1.696 V, while the latch-down voltage increased from 1.486 to 1.637 V. Mechanisms of temperature-dependent change in latch voltage were analyzed using energy band diagrams. This temperature-dependent analysis on silicon biristor can serve as blueprint for the contribution of stable operation.

## 1. Introduction

The one-transistor dynamic random-access memory (1T-DRAM) [1] has the potential to make device manufacturing easier and implement higher integration compared with existing DRAMs. However, the main disadvantage of 1T-DRAMs is the gate dielectric degradation induced by hot-carrier injection [1,2,3]. To solve this problem, bistable resistors (biristors) have been proposed for two-terminal memory devices [2,3,4]. The biristor is an open-base bipolar junction transistor that is equivalent to a gateless metal-oxide-semiconductor field-effect transistor [2,4,5,6]. Because of its gateless structure, the problem of a three-terminal structure has been solved for applications in various fields [7]. The biristor has two stable resistance states and exhibits hysteretic current-voltage characteristics [2]. Therefore, this device can be used as a memory device [8]. The biristor can also be used as a current pulse generator with a high current rise rate; as a lighting trigger switch through an optical response; and a biosensor through electrical detection [2,9]. Recently, the use of a biristor as a leaky integrate-and-fire neuron in neuromorphic systems has been proposed [10,11].

Despite the outstanding electrical characteristics of biristors, their high operating voltage hinders their use as replacements for conventional memory devices. Several studies have been conducted to increase the current gain and decrease the operating voltage of biristors. A typical method to increase the current gain is the use of silicon germanium (SiGe) [12]. A bandgap-engineered SiGe biristor can reduce the latch-up voltage using the heterogeneous bandgap structure [5]. This also expands the latch voltage window, which is the difference between the latch-up and latch-down voltage [5]. Vertical InGaAs biristors have a lower operating voltage than SiGe biristors and can be used in 3D integrated applications such as stacked neuron devices in artificial neural networks [13]. Another method to increase the current gain is through the surface accumulation layer transistor (SALTran) [14,15], which can decrease the operating voltage without using heterojunctions or other complex processes [16]. Although the current gain is dependent on the temperature, no in-depth studies have been conducted on the temperature-dependent electrical characteristics of biristors [17]. Temperature-dependent analysis of the biristor is important to maintain the stable operation of the memory circuit. In this study, the operating mechanism of a silicon SALTran biristor in the temperature range of 275–400 K is investigated.

## 2. Device Structure and Simulation

**Figure 1:**
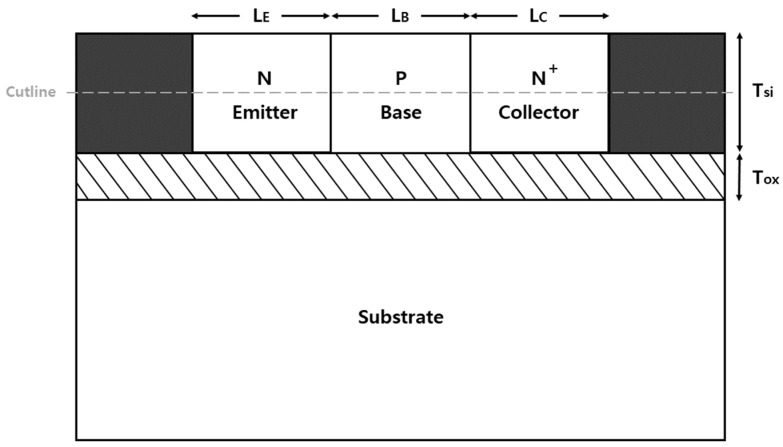
Cross-section of the biristor with cutline.

The cross-sectional view of the biristor used in this study is shown in Figure 1. The study was performed on a two-dimensional structure for simulation purposes using a device simulator (Silvaco Atlas, version 5.2.17. R) [18]. In designing the biristor, silicon-on-insulator technology was used to preserve the excess holes caused by impact ionization [2]. To obtain a high current gain, a metal contact with a work function less than that of Si and a lightly doped emitter were used in the biristor [16]. This method uses the SALTran concept.

According to previous studies [16], electrons accumulated by low work function metal contact are unevenly distributed, resulting in the formation of an electric field near the metal-emitter contact interface. The direction of the induced electric field is opposite to the flow of holes from the base into the emitter. This reduces the gradient of the excess hole, but the gradient of the excess electrons injected into the base from the emitter is not affected. The current gain is improved by leading to a decrease in the base current for the collector current. Accordingly, the current gain of the SALTran biristor (surface accumulation layer transistor bistable resistor) is improved so that the latch-up voltage and the latch-down voltage are reduced. In the case of the proposed device, the base length was reduced to 30 nm by controlling the doping concentration in the existing SALTran biristor.

The dimensional parameters include an emitter length (L_E_) of 30 nm, a base length (L_B_) of 30 nm, collector length (L_C_) of 30 nm, silicon film thickness (T_Si_) of 40 nm, and silicon dioxide layer thickness (T_OX_) of 15 nm. The width of the device in the Z-direction is 1 μm. The emitter, base, and collector had doping concentrations of 4 × 10^12^ cm^−3^, 5 × 10^18^ cm^−3^, and 1 × 10^20^ cm^−3^. Aluminum was used for the emitter/collector electrodes. The emitter/collector electrode work function was 3.9 eV. It was set as a parameter with a high current gain in order to show a lower operating voltage than a conventional biristor. The current gain was increased by reducing the base length and emitter length and lowering the emitter doping concentration. The models used in the Atlas device simulation [17] include the trap -assisted tunneling model, Masetti low-field mobility model, parallel electric-field-dependent mobility model, concentration-dependent Shockley–Read–Hall model, bandgap narrowing model, energy balance model, and Toyabe impact ionization model. The basic parameters were applied in all the models. In this study, all biristor simulations were performed in the temperature range of 275–400 K. At 25 K intervals from 275 K to 400 K, forward and reverse sweeps were conducted at each temperature to observe the change in the biristor.

## 3. Results and Discussion

**Figure 2:**
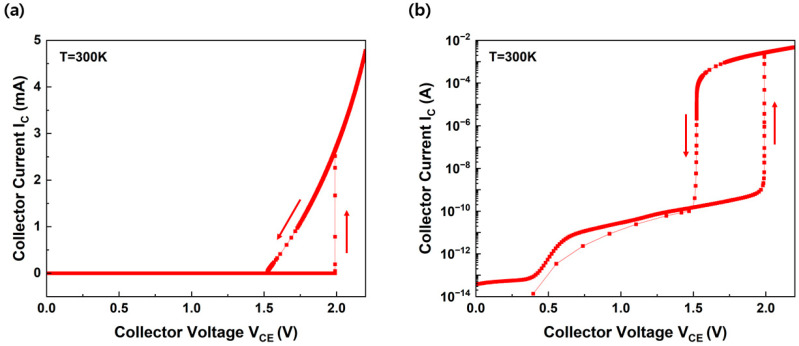
Hysteresis of the I−V characteristics of the biristor on the (**a**) linear scale and (**b**) log scale. Arrows indicate sweeping directions.

The hysteresis current-voltage (I–V) characteristics of the biristor at 300 K on the linear and logarithmic scales are shown in Figure 2a and 2b, respectively. The collector current gradually increased as the collector voltage was swept forward. The collector current (*I_C_*) was amplified from the current gain (β), multiplication factor (M), and base current (*I_B_*) [19], expressed as

$$I_{C}=\frac{M\times \beta }{1-(M-1)\times \beta }I_{B}$$

When the collector voltage approaches the latch-up voltage, impact ionization occurs, resulting in a latch-up that rapidly increases the current. The latch-up voltage is defined as the collector voltage when impact ionization triggers an open-base breakdown [20]. That is, when the voltage level satisfies (M − 1) × β = 1, the abrupt increase in the current corresponding to the latch-up phenomenon occurs. This device maintained a latch-up state despite the further increase in voltage. In other words, after latch-up occurred, the biristor was turned on. During a reverse collector voltage sweep, the collector current decreases. When the collector voltage approaches the latch-down voltage, the impact ionization decreases, resulting in a latch-down in which the current rapidly decreases. The latch-down voltage is the collector voltage when the open-base destruction is suppressed [20]. The device does not turn off until the applied voltage falls below the latch-down voltage. When the voltage is less than the latch-down voltage, the biristor turns off. This indicates that the latch-up voltage of the forward sweep is higher than that of the reverse sweep. The M and β values, which are important parameters in the positive-feedback process, vary depending on the presence of excessive holes [8]. Therefore, the biristor exhibits bistable I–V characteristics between the latch-up and latch-down voltages. The latch characteristics are significantly affected by the base length [5,21]. According to previous studies, the latch-up and latch-down voltages decreased as the base length narrowed, but the latch window became very small as the off-state current increased [13]. Our biristor exhibited a latch-up voltage, latch-down voltage, and latch window of 1.990 V, 1.522 V, and 0.468 V, respectively, with a base length 30 nm shorter than that of a conventional biristor.

**Figure 3:**
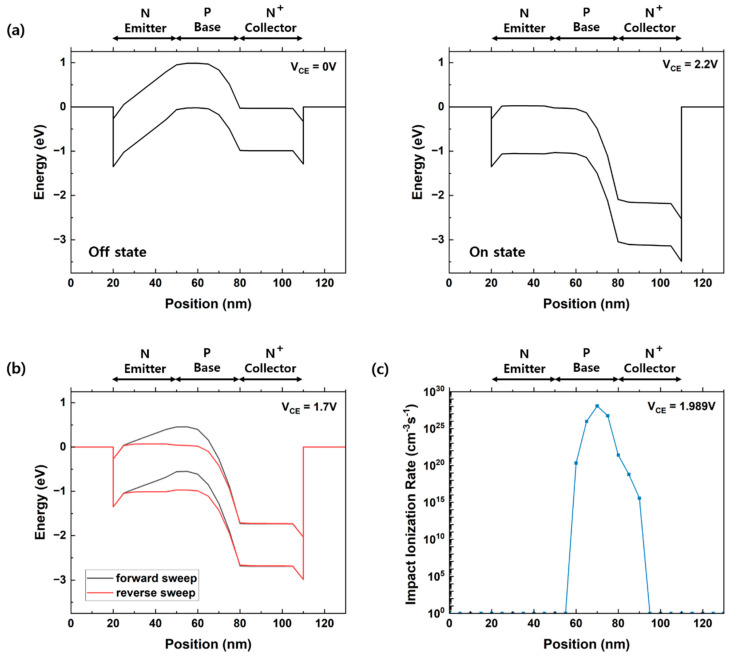
(**a**) Energy−band diagram of the biristor in OFF and ON states. (**b**) Energy−band diagram of biristor biased to V_CE_ = 1.7 V during forward/reverse sweeps. (**c**) Impact ionization rate of the biristor biased at the latch-up voltage (T = 300 K).

Figure 3 was extracted along the cutline of the biristor shown in Figure 1. Figure 3a shows the on/off states of the biristor before and after latch-up occurred at 300 K in a band diagram. In the off-state, the potential barrier of the base region blocked the flow of electrons from the collector; hence, no current flowed. When a low collector voltage was applied, the collector current was determined by drift and diffusion. A positive-feedback process was generated when the applied voltage increased sufficiently to cause impact ionization. When impact ionization was induced at the base collector junction, electron-hole pairs were generated. The p-type base region served as a potential well. Holes accumulated in the potential well, which electrically reduced the height of the potential barrier in the base area and provided more electrons for impact ionization. An increase in the number of holes injected into the base area further increased the body potential, causing a positive-feedback phenomenon that turned the biristor on. Unlike impact ionization field-effect transistors which operate by impact ionization [22], our device operates by the positive feedback phenomenon generated by impact ionization. Although the decreasing device size may weaken the impact ionization effect, the device can stably operate. The positive-feedback phenomenon persisted until the negative feedback phenomenon began. The decrease in the applied voltage stimulated the recombination of electrons and holes. The carrier accumulated in the potential wells was removed. As a result, the potential barrier at each junction gradually increased. When the input voltage was reduced to below the latch-down voltage, the potential barrier was reproduced by the negative feedback phenomenon, and the biristor was turned off. The energy-band diagram at V_CE_ = 1.7 V during forward and reverse sweeps is shown in Figure 3b. The potential barrier was controlled by the excess holes. In the case of the forward sweep, the potential barrier height in the base region was high before the occurrence of positive feedback; therefore, electrons could not be injected into the collector. However, in the reverse sweep, 1.700 V was re-applied after the biristor was turned on; hence, the potential barrier height in the base region decreased and could be injected into the collector. Figure 3c shows the impact ionization rate of the biristor at the latch-up voltage. The occurrence of impact ionization indicated that excess holes were generated in the base region. Accordingly, a positive-feedback phenomenon occurred, making it possible to turn on the biristor.

**Figure 4:**
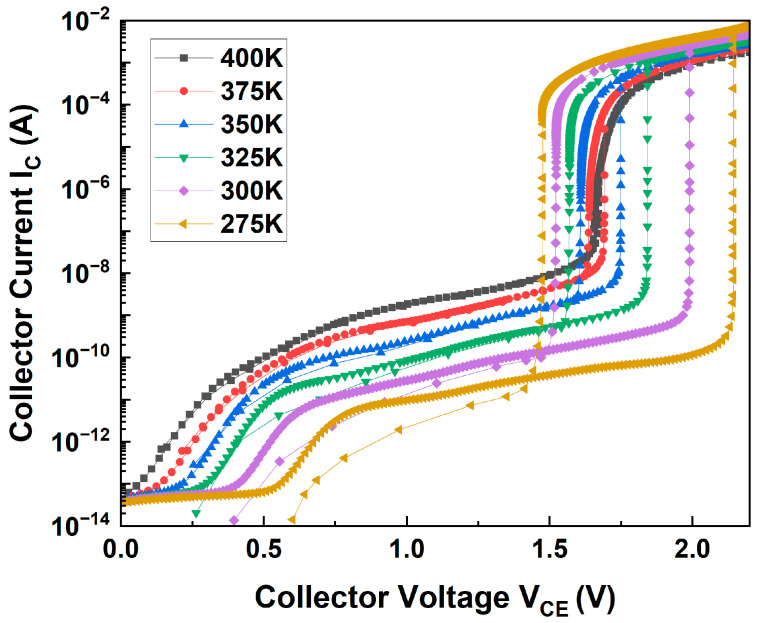
Simulated temperature-dependent hysteretic I–V characteristics of the biristor.

Figure 4 shows the hysteretic I-V characteristics of the biristor at 275–400 K. The phenomenon is similar to that of the conventional biristor, but latch-up and latch-down occurred at lower voltages than the conventional biristor [23]. As the temperature increased from 275 K to 375 K at intervals of 25 K, the latch-up voltage decreased, while the latch-down voltage increased (Table 1). The latch voltage is affected by the current gain. Carrier lifetime depends on the doping concentration and temperature [24]. The following equation represents the electron lifetimes are expressed as

$$\tau_{n}=\frac{\tau_{max,n}{\left(\frac{T}{300}\right)}^{\alpha }}{1+{\left(\frac{N_{i}}{3\times 10^{17}}\right)}^{\gamma }}$$

The increase in the carrier lifetimes tends to increase the current gain [24]. As the temperature increases, the carrier lifetime increases. Therefore, the current gain increases, resulting in a lower latch-up voltage and a higher latch-down voltage. The change in temperature also led to changes in the carrier concentration and potential barrier. As the temperature increased, the carrier concentration increased; thus, the potential barrier increased. As a result, the latch voltage window, which is the difference between the latch-up and latch-down voltages, decreased as the temperature increased (0.668 → 0.468 → 0.274 → 0.142 → 0.054 → 0.012 V). In addition, the increase in temperature increased the leakage current; hence, the latch phenomenon did not occur at 400 K because it interrupted the positive-feedback process.

**Figure 5:**
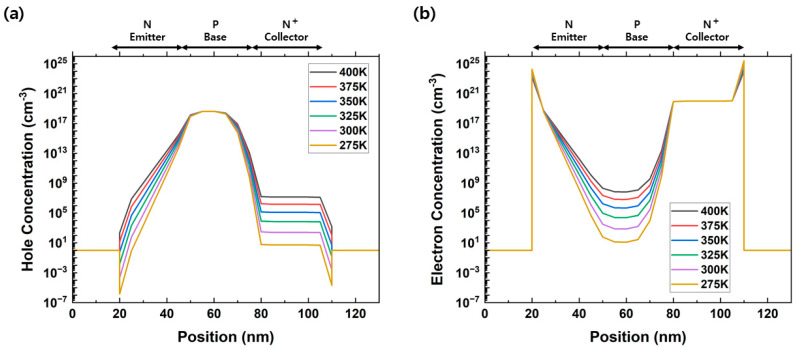
(**a**) Hole and (**b**) electron concentrations with increasing temperature.

Figure 5a,b show the changes in hole and electron concentrations according to the temperature, respectively. These data were extracted according to the cutline of the biristor. As the temperature increased, the hole concentrations in the emitter and collector region, and the electron concentrations in the base region increased. The increase in hole and electron concentrations reduced the height of the potential barrier, which plays an important role in determining the latch-up/latch-down voltage. The collector voltage required to trigger the positive-feedback loop was relatively low because of the decrease in the potential barrier height. That is, as the temperature increased, the latch-up voltage decreased and the latch-down voltage increased. These results indicate that the operating voltage decreased owing to the increase in the common emitter gain (β). The increased electron concentration in the doped p-type base region increased the common emitter gain.

**Figure 6:**
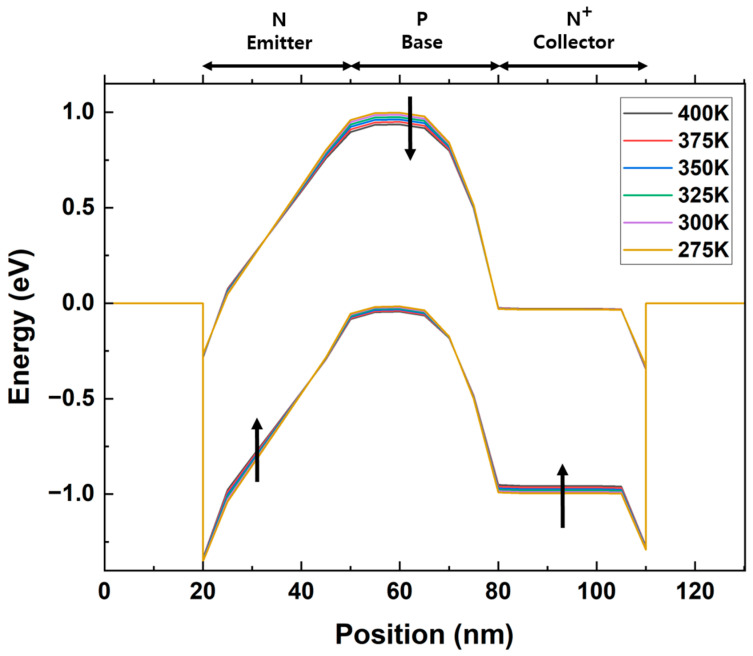
Variations in energy bands of the biristor with increasing temperature. Arrows indicate the corresponding energy band diagrams from 275 K to 400 K.

Figure 6 is extracted along the cutline and shows the energy-band diagram of the biristor as the temperature increases. In the emitter and collector regions, the valence band gradually shifted upward as the temperature increased from 275 K to 400 K at 25 K intervals. The corresponding energy levels are −0. 663 → −0.658 → −0.653 → −0.648 → −0.643 → −0.636 eV in the emitter region and −0.995 → −0.981 → −0.973 → −0.955 → −0.957 eV for the collector region. In contrast, the potential barrier that formed in the conduction band in the base region gradually decreased as the temperature increased (0.978 → 0.967 → 0.955 → 0.942 → 0.930 → 0.917 eV). Carrier concentration and energy gap are inversely proportional according to the following equation.

$$\begin{array}{l} n=N_{c}e^{-\left(E_{c}-E_{Fn}\right)∕kT}\\ p=N_{v}e^{-\left(E_{Fp}-E_{v}\right)∕kT} \end{array}$$

The energy gap decreases due to the increase in carrier concentration as the temperature increases. The energy gap in the base region decreased, thereby allowing a lower bandgap to increase the impact ionization activity. As the temperature increased, more impact ionization occurred, and the height of the potential barrier decreased. Therefore, the decrease in the potential barrier height decreased the latch-up voltage and increased the latch-down voltage.

## 4. Conclusions

In this study, the temperature-dependent electrical characteristics of silicon biristors were investigated through simulation. At room temperature, silicon biristors, which employed asymmetrical emitters and collector doping to increase the current gain, had lower operating voltages compared with those of previously reported silicon biristors [2,4,6]. The biristor was turned on and off by positive and negative feedback phenomena. As the temperature increased from 275 K to 375 K, the latch-up voltage decreased from 2.144 V to 1.691 V, and the latch-down voltage increased from 1.476 V to 1.637 V. That is, as the temperature increases, the latch window decreases. Increasing temperature leads to an increase in hole and electron concentrations and more impact ionization rates. This causes a decrease in the height of the potential barrier, resulting in a decrease in the latch window. Such a biristor temperature-dependent analysis may contribute to stable operation in a memory circuit. The proposed device can also help with artificial neuron research. The configuration of artificial neurons is important for the hardware implementation of SNN (spiking neural network) [10]. The biristor may contribute to research on artificial neurons at the device level for area efficiency. Research to confirm the performance and reliability of SALTran biristor can be used as neuron devices, or studies such as long-term stability tests, search at wider temperatures, and integration into more complex circuits can be conducted.

## Figures and Tables

**Table 1:** Variation of latch-up and latch-down voltages depending on temperature.

Temperature	275 K	300 K	325 K	350 K	375 K	400 K
**Latch-up voltage (V)**	2.144	1.990	1.842	1.749	1.691	1.667
**Latch-down voltage (V)**	1.476	1.522	1.568	1.607	1.637	1.655

## Data Availability

Data are contained within the article.

## References

[1] Okhonin S., Nagoga M., Sallese J.M., Fazan P. (2002). A capacitor-less 1T-DRAM cell. IEEE Electron Device Lett..

[2] Han J.-W., Choi Y.-K. (2010). Biristor—Bistable Resistor Based on a Silicon Nanowire. IEEE Electron Device Lett..

[3] Aoulaiche M., Collaert N., Degraeve R., Lu Z., De Wachter B., Groeseneken G., Jurczak M., Altimime L. (2010). BJT-Mode Endurance on a 1T-RAM Bulk FinFET Device. IEEE Electron Device Lett..

[4] Han J.-W., Choi Y.-K. Bistable resistor (biristor)—Gateless silicon nanowire memory. Proceedings of the 2010 Symposium on VLSI Technology.

[5] Moon J.-B., Moon D.-I., Choi Y.-K. (2014). A Bandgap-Engineered Silicon-Germanium Biristor for Low-Voltage Operation. IEEE Trans. Electron Devices.

[6] Moon D.I., Choi S.J., Kim S., Oh J.S., Kim Y.S., Choi Y.K. (2011). Vertically Integrated Unidirectional Biristor. IEEE Electron Device Lett..

[7] Moon D.I., Choi S.J., Kim J.Y., Ko S.W., Kim M.S., Oh J.S., Lee G.S., Kang M.H., Kim Y.S., Kim J.W. Highly endurable floating body cell memory: Vertical biristor. Proceedings of the 2012 International Electron Devices Meeting.

[8] Lim D., Kim M., Kim Y., Cho J., Kim S. (2018). Nondestructive Readout Memory Characteristics of Silicon Nanowire Biristors. IEEE Trans. Electron Devices.

[9] Moon D.I., Peycelon M., Kim J.Y., Ahn J.H., Jung Park T., Choi Y.K. (2013). A biristor based on a floating-body silicon nanowire for biosensor applications. Appl. Phys. Lett..

[10] Han J.-W., Meyyappan M. (2018). Leaky Integrate-and-Fire Biristor Neuron. IEEE Electron Device Lett..

[11] Han J.-K., Yun S.-Y., Lee S.-W., Yu J.-M., Choi Y.-K. (2022). A Review of Artificial Spiking Neuron Devices for Neural Processing and Sensing. Adv. Funct. Mater..

[12] Kwok K.H., Selvakumar C.R. (2001). Profile design considerations for minimizing base transit time in SiGe HBTs for all levels of injection before onset of Kirk effect. IEEE Trans. Electron Devices.

[13] Kim W.K., Bidenko P., Kim J., Sim J., Han J.K., Kim S., Geum D.M., Kim S., Choi Y.K. (2021). Vertical InGaAs Biristor for Sub-1 V Operation. IEEE Electron Device Lett..

[14] Kumar M.J., Parihar V. (2004). Surface accumulation Layer transistor (SALTran): A new bipolar transistor for enhanced current gain and reduced hot-carrier degradation. IEEE Trans. Device Mater. Reliab..

[15] Kumar M.J., Singh P. (2006). A super beta bipolar transistor using SiGe-base surface accumulation layer transistor(SALTran) concept: A simulation study. IEEE Trans. Electron Devices.

[16] Kumar M.J., Maheedhar M., Varma P.P. (2015). A Silicon Biristor With Reduced Operating Voltage: Proposal and Analysis. IEEE J. Electron Devices Soc..

[17] Nr S., Singh S., Kumar P. (2021). Si1−xGex nanowire based metal-semiconductor-metal Schottky biristor: Design and sensitivity analysis. IET Circuits Devices Syst..

[18] Silvaco (2004). Silvaco User’s Manual Device Simulation Software.

[19] Reisch M. (1992). On bistable behavior and open-base breakdown of bipolar transistors in the avalanche regime-modeling and applications. IEEE Trans. Electron Devices.

[20] Kim D.-O., Moon D.-I., Choi Y.-K. (2014). Optimization of Bias Schemes for Long-Term Endurable 1T-DRAM through the Use of the Biristor Mode Operation. IEEE Electron Device Lett..

[21] Son J.W., Hur J., Kim W.-K., Lee G.-B., Choi Y.-K. (2020). A Strategy for Optimizing Low Operating Voltage in a Silicon Biristor. IEEE Trans. Nanotechnol..

[22] Gopalakrishnan K., Griffin P.B., Plummer J.D. I-MOS: A novel semiconductor device with a subthreshold slope lower than kT/q. Proceedings of the Digest. International Electron Devices Meeting.

[23] Han J.W., Meyyappan M. (2014). Trigger and self-latch mechanisms of n-p-n bistable resistor. IEEE Electron Device Lett..

[24] Li X., Luo Y., Fursin L., Zhao J.H., Pan M., Alexandrov P., Weiner M. (2003). On the temperature coefficient of 4H-SiC BJT current gain. Solid-State Electron..

